# Transcranial Electromagnetic Wave Treatment: A Fountain of Healthy Longevity?

**DOI:** 10.3390/ijms24119652

**Published:** 2023-06-02

**Authors:** Gary Arendash, Chuanhai Cao

**Affiliations:** 1 NeuroEM Therapeutics, Inc., 501 E. Kennedy Blvd., Suite 650, Tampa, FL 33602, USA; 2 Taneja College of Pharmacy, University of South Florida, Tampa, FL 33612, USA; ccao@usf.edu; 3 MegaNano Biotech, 3802 Spectrum Blvd., Suite 122, Tampa, FL 33612, USA

**Keywords:** Transcranial Electromagnetic Wave Treatment, health span, longevity, inflammation, cytokines, immune imbalance

## Abstract

Most diseases of older age have as their common denominator a dysfunctional immune system, wherein a low, chronic level of inflammation is present due to an imbalance of pro-inflammatory cytokines over anti-inflammatory cytokines that develops during aging (“inflamm-aging”). A gerotherapeutic that can restore the immune balance to that shared by young/middle-aged adults and many centenarians could reduce the risk of those age-related diseases and increase healthy longevity. In this perspectives paper, we discuss potential longevity interventions that are being evaluated and compare them to a novel gerotherapeutic currently being evaluated in humans—Transcranial Electromagnetic Wave Treatment (TEMT). TEMT is provided non-invasively and safety through a novel bioengineered medical device—the MemorEM—that allows for near complete mobility during in-home treatments. Daily TEMT to mild/moderate Alzheimer’s Disease (AD) patients over a 2-month period rebalanced 11 of 12 cytokines in blood back to that of normal aged adults. A very similar TEMT-induced rebalancing of cytokines occurred in the CSF/brain for essentially all seven measurable cytokines. Overall inflammation in both blood and brain was dramatically reduced by TEMT over a 14–27 month period, as measured by C-Reactive Protein. In these same AD patients, a reversal of cognitive impairment was observed at 2 months into treatment, while cognitive decline was stopped over a 2½ year period of TEMT. Since most age-related diseases have the commonality of immune imbalance, it is reasonable to postulate that TEMT could rebalance the immune system in many age-related diseases as it appears to do in AD. We propose that TEMT has the potential to reduce the risk/severity of age-related diseases by rejuvenating the immune system to a younger age, resulting in reduced brain/body inflammation and a substantial increase in healthy longevity.

## 1. Introduction

Most people would greatly desire living a longer life with fewer age-related diseases—in other words, increasing their “health span”. To this end, “healthy longevity” has become a new and exciting 21st century focus, though still in its infancy. Unfortunately, no therapeutic intervention has yet been shown to increase the human health span. For most companies/institutions involved in this research area, the four main approaches being investigated to possibly increase healthy longevity or to develop rejuvenating interventions therein are:

(1) Trying to get rid of senescent cells in the body to limit inflammation [1];
(2) Experimenting with supplements for diseases of aging, such as Nicotinamide (NAD) precursors [2];
(3) Building databases of human genotypes in an effort to develop genetic interventions (ClinicalTrial.gov NCT00707694; accessed on 17 April 2023);
(4) Studying animal models of aging to try to understand how they may live longer through various interventions [3,4].

Certainly, these approaches have yielded valuable insight into longevity from pre-clinical studies. However, they continue to be at an early stage in development for trying to find a therapeutic intervention that can increase the human health span. To that end, there are a number of “gerotherapeutic” drugs/compounds currently being investigated for increasing healthy longevity, with the following four being of primary focus:

(1) **Methformin**: Methformin has been used for many years to treat type 2 diabetes. In addition to its anti-hyperglycemic effect, it appears able to indirectly suppress pro-inflammatory pathways. Methformin has a long record of clinical trials purporting to reduce cancer, cognitive impairment, and mortality [5], apparently indirectly through its antihyperglycemic and insulin-sensitizing effects [6]. A completed clinical trial (Methformin in Longevity Study; MILES) administered Methformin or placebo for 6 weeks to normal aged subjects, resulting in reduced gene expression in muscle and fat tissue [7]. A second clinical trial (Targeting Aging with Methformin; TAME), not yet started, is being planned to administer Methformin or placebo for 6 years to normal individuals aged 65–80 years old, with endpoints of age-related disease occurrence and biomarkers of aging [6].
(2) **Nicotinamide (NAD) Precursors:** As a naturally-occurring metabolite, NAD is involved in redox reactions and cellular energy production. Since it becomes reduced in many tissues during aging [8,9], precursors such as nicotinamide riboside and nicotinamide mononucleotide are being evaluated pre-clinically.
(3) **Rapamycin**: Rapamycin is an FDA-approved immunosuppressive drug used primarily to prevent organ allograft rejection. A small controlled clinical trial administering Rapamycin to healthy aged subjects for eight weeks did not induce any changes in cognitive performance, immune endpoints, or self-perceived health [10].
(4) **Dasatinib+Quercetin, Fisetin**: Senescent cells are cells that undergo age-related changes and/or have stopped dividing, after which they begin producing an array of pro-inflammatory cytokines and growth factors (called the Senescence-Associated Secretory Phenotype; SASP). These pro-inflammatory secretions from senescent cells are thought to contribute to the increased inflammation (“inflamm-aging”) in body and brain during aging [11]. Senolytics such as Dasatinib+Quercetin (DQ) and Fisetin search out and attempt to destroy such senescent cells to reduce inflammation [1]. Among the clinical trials of Dasatinib+Quercetin and/or Fisetin that have been planned or are on-going are those involving kidney disease, osteoporosis, osteoarthritic, and early AD [12,13]. Regarding early AD, a pilot clinical trial of five early AD subjects being given DQ for 3 months was recently completed, which reported no beneficial cognitive effects and no changes in AD markers in plasma or brain post-treatment [14]. Although the number of clinical trials involving senolytics is increasing, there have been no clinical trials to our knowledge showing that any senolytic drug can delay, stop, or reverse the main characteristics of an age-related disease or enhance lifespan.

We believe the aforementioned approaches and clinical interventions therein, though scientifically valid, will not lead to a wide-ranging breakthrough for a human “Fountain of Healthy Longevity” anytime soon. Rather, we believe that finding such a fountain for humans requires analyses and clinical studies in extremely-aged humans, as some longevity researchers have been doing in attempting to identify gene variants that are linked to longer lifespans and that could be targeted by drugs [15]. More directly, longevity research should be focused on learning how and why humans who live 100+ years (centenarians) have lived so long, often in good health [16]. However, such studies involving long-lived human populations in the “Blue Zone” (e.g., Okinawa, Sardinia, Ikaria) have often been centered on lifestyle or environmental factors, rather than on the physiological characteristics that are associated with their extreme longevity [17].

It appears that no more than 25% of human longevity factors directly involve genetics, with some estimates being as low as 10–16% [18]. So, the other 75–90% can presumably be modified to increase the lifespan. Lifestyle (e.g., healthy diet, exercise, smoking, drugs) certainly contributes some to this 75–90%, but there must be another more overriding factor that increases lifespan, otherwise there would be many more centenarians than there currently are.

## 2. Following the Pathway of Centenarians to Healthy Longevity

So, the critical question is: Aside from possible genetic advantages, *what is it about Centenarians that provides for their extreme longevity*? From a variety of studies, the answer appears to be that many centenarians have a “balanced” immune system made up of robust pro- and anti-inflammatory components, as indexed by the immune system’s effectors—its cytokines [19,20,21]. Centenarians have gene variants that allow them to optimize the resilience and balance between pro- and anti-inflammatory cytokines, thus minimizing the effects of lifelong exposure to environmental and lifestyle insults [15,22]. In other words, centenarians have a robust pro-inflammatory component, as well as a balanced and equally robust anti-inflammatory component, with both working together to effectively provide centenarians with healthy longevity. Along this line, a recently-developed “inflammatory clock of aging” was used to determine the inflammatory ages of 29 centenarians and found that they had inflammatory ages averaging 40 years less than their calendar age [23].

Immune balance is present in humans through middle age, but is thereafter lost as one goes into older age [24]. After middle age, the immune system’s pro-inflammatory cytokines become more and more prominent over anti-inflammatory cytokines, resulting in continual, low-grade “inflamm-aging” in the brain and body that causes or is involved with most diseases of aging [25,26]. Inflammaging involves persistently elevated levels of pro-inflammatory cytokines capable of activating both the innate and adaptive immune systems [21]. For example, plasma levels of pro-inflammatory cytokines have been reported to increase 2–4 fold during aging [27] and serum levels of multiple cytokines such as GCSF, GMCSF, and TGF-α are increased in subjects over 65 compared to young and middle-aged subjects [28]. Even in the brain, levels of the cytokines TNF-α, IL-8, and IL-10 gradually increase during aging, as indexed by their levels in the brain’s cerebrospinal fluid (CSF) [29].

Since both pro- and anti-inflammatory immune components stay strong through the lives of centenarians, we believe an intervention that can maintain or re-establish immune system balance, wherein both pro- and anti-inflammatory components stay strong and body/brain inflammation is minimized, will provide the immune profile seen in centenarians—thus resulting in an increase in lifespan with fewer age-related diseases occurring, as enjoyed by centenarians. Indeed, the overall strategy in targeting inflammaging is shifting from trying to inhibit inflammation (i.e., suppressing pro-inflammatory cytokines) to seeking interventions that re-establish a balanced immune system of cytokines [20]. Supportive of that new strategy, mice that maintain a youthful balance of cytokines systemically are “extremely long-lived” [30].

If a dysfunction (e.g., unbalanced immune system) is common to most diseases of aging, it is plausible that a therapeutic intervention that stops or reverses the manifestations of a single major disease of aging through immune system rebalancing could be successful at treating multiple other age-related diseases. A major age-related disease is Alzheimer’s Disease, which involves an imbalanced cytokine profile generally evident as low-grade inflammation in the brain and body [20,31,32]. Thus, AD should serve as a good test for any gerotherapeutic intervention being presented as capable of “increasing health longevity”. We believe such a gerotherapeutic may be a new bioengineered technology we have developed—Transcranial Electromagnetic Wave Treatment (TEMT). In this perspectives paper, we provide rationale for the use of TEMT to rebalance the out-of-balance immune system common to most diseases of aging and, in so doing, decrease disease risk/severity to increase healthy longevity.

## 3. Transcranial Electromagnetic Wave Treatment: Effects on Longevity, Cognition, AD Markers, Brain Imaging, and Mechanisms of Action

Given the failure of pharmaceutical interventions against multiple neurological diseases of aging, neuromodulatory approaches have surfaced as an alternative treatment strategy. These non-pharmacological approaches include repetitive transcranial magnetic stimulation (rTMS), transcranial alternating current stimulation (tACS), transcranial ultrasound (tUS), and LED/IR light stimulation. They all have neuronal stimulation as their mechanism of action. For example, rTMS involves a magnetic coil (magnet) placed close to the subject’s head, with an electrical (AC) current passed through the coil to generate a circulating magnetic field, which then induces a perpendicular electric field within the subject’s head to stimulate neurons. These magnetic and electric fields dissipate as they go through the cerebral cortex and consequently do not generally affect sub-cortical brain structures. rTMS is often referred to as “electromagnetic stimulation”, but that is misleading because true electromagnetic “waves” are not generated by the magnets of rTMS (see next paragraph). As such, the mechanism of rTMS action is different from TEMT in primarily involving a rTMS frequency-dependent stimulation (depolarization) or inhibition (hyperpolarization) of neurons in the cerebral cortex [33]. More specifically, rTMS appears to alter synaptic plasticity in the cerebral cortex through long-term potentiation/depression (LTP/LTD) of excitatory synaptic transmission [34], resulting in changes in neurotransmitter levels, functional connectivity, and various molecular pathways [33]. Although rTMS has been successfully used to treat depression [35,36] and obsessive-compulsive disorder [37,38], its ability to treat diseases of aging has yet to be established. Regarding AD, the largest rTMS study to date was a multi-site controlled Phase III study in 2020 involving 131 AD subjects given tMS treatment or not for 6 weeks [39]. No cognitive benefits of rTMS were observed following this 6-week stimulation period, although other smaller uncontrolled studies did report cognitive benefits [40,41]. Issues thus far identified in clinical studies of rTMS in age-related diseases in general, and AD in particular, are that: (1) rTMS may actually aggravate disease development through disruption of excitatory/inhibitory balance in the brain, (2) rTMS studies have thus far largely not measured markers of aging/AD in the brain or blood, and (3) rTMS studies are generally less than a couple of months [40]. In addition, the various parameters of rTMS (i.e., brain area targeted, use of high or low frequency rTMS, duration of treatment) may all impact whether or not beneficial effects may be seen in age-related diseases such as AD.

**Figure 1:**
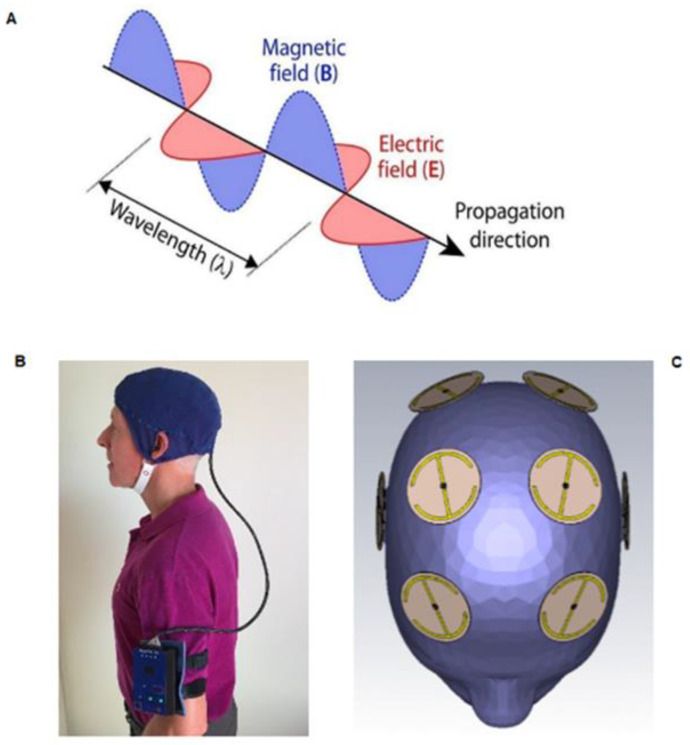
(**A**) Electric and magnetic waves, at right angles to one another, comprising electromagnetic waves; (**B**) the MemorEM device being worn by a subject, allowing near complete mobility during in-home treatments; (**C**) location of the eight electromagnetic wave emitters between the two-layered head cap.

We have pioneered a new bioengineering-based technology, Transcranial Electromagnetic Wave Treatment (TEMT), which is very different from the aforementioned neuromodulatory approaches in having demonstrated multiple mechanisms of action that do not involve neuronal stimulation. For humans, TEMT involves the generation of electromagnetic (radiofrequency) waves by an electromagnetic wave generator. These EMF waves radiate away from emitters, never to return, which is in contrast to the circulating magnetic fields of rTMS. Moreover, EMF waves actually consist of linked sinusoidal electric and magnetic “waves”, generating an electric field, magnetic field, and direction of wave propagation all aligned perpendicular to each other (Figure 1A). Such electromagnetic fields (EMFs) can be generated by a small EMF generator worn on the arm, which is connected via wires to multiple (eight) emitters within a double-layered cap that are positioned to provide full-brain EMF treatment (Figure 1B,C). The unique device we have developed for such treatment, the *MemorEM*, is fully self-contained and allows for near complete in-home mobility during daily 1 h treatments, as self-administered by the subject or by the subject’s caregiver. Emitters are “ON” sequentially at 217 Hz, at a radiofrequency of 915 MHz and a power level of 1.6 W/kg SAR. These specific parameters were used so successfully in our pre-clinical studies involving Alzheimer’s transgenic and normal mice [42,43,44,45,46,47] that we have continued their use in our ensuing clinical studies. As described later in Section 3, there are three primary mechanisms of TEMT action that we have identified: disaggregation of toxic protein oligomers in the brain, mitochondrial enhancement, and rebalancing of an unbalanced immune system.

There is significant evidence, not associated with any particular mechanism(s), that TEMT/EMF treatment at around 900–915 MHz can increase longevity and decrease age-related diseases. Examples of this evidence are:

(1) A 15–20% increase in cell survival occurred in N2a/APPswe cell cultures given twice-daily EMF treatment compared to controls (Cao, being submitted).
(2) With life-long EMF treatment, twice as many rats survived to two years of age vs. control rats, with survival probabilities of 0.50 and 0.28, respectively [48].
(3) Glioma (brain cancer) patients exposed to TEMT had increased survival in comparison to glioma patients having no exposure [49].
(4) A 30–40% reduction in hospitalizations due to Alzheimer’s Disease, all other types of dementia, and Parkinson’s Disease was observed for subjects having long-term TEMT exposure [50].
(5) During a 2½-year period of TEMT in AD subjects averaging 71 years of age, no new incidence of any age-related diseases (e.g., neurological diseases, cancer, arthritis) occurred [51].

In as much as AD is clearly an age-related neurodegenerative disease that affects longevity, much of this paper will present our three studies of clinical work in AD subjects [51,52,53] and ancillary literature supporting our view that TEMT should address many diseases of aging to extend the human lifespan.

**Figure 2:**
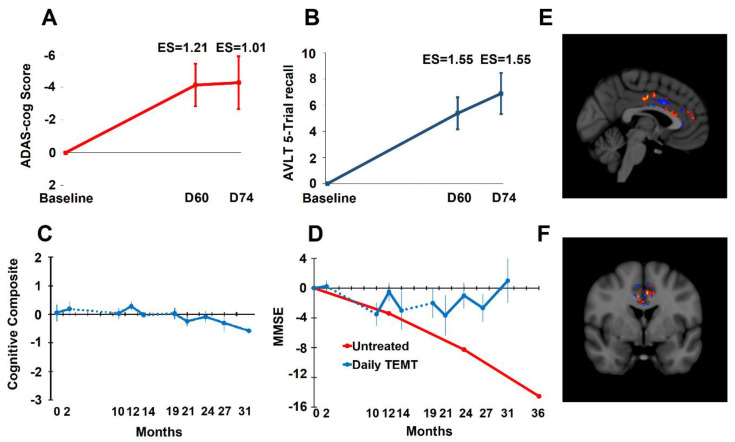
(**A**,**B**) TEMT reverses Alzheimer’s memory impairment in ADAS-cog13 and Rey AVLT 5-Trial Recall. Improvement after 60 days of TEMT was maintained for at least 2 weeks after TEMT. Red and blue lines represent the same AD patients tested in both tasks; ES = effect size (as Cohen’s d) compared to baseline. An ES greater than 0.8 and 1.20 indicates clinically significant effects that are large and very large, respectively. (**C**) Over a period of 2½ years, AD patients given TEMT showed no “overall” cognitive decline in a Cognitive Composite score made up of ADAS-cog13, Rey AVLT, MMSE, Digits Forward, Digits Backward, and ADL; *p* = 0.399 for change-over-time mixed effects analysis; *p* = 0.328, t = 1.28 for baseline vs. 31 months. (**D**) The MMSE component of the Cognitive Composite score showed no significant decline in MMSE scores over this same extended 2½ year period (for change-over-time mixed effects analysis, *p* = 0.266; for baseline vs. 31 months, *p* = 0.287, t-1.44). Dotted lines indicate the 8-month and 5-month periods of no treatment. The red line and data points are from another study [54] evaluating MMSE longitudinally in a group of “untreated” mild/moderate AD subjects with near identical demographics and baseline MMSE scores. (**E**,**F**) fMRI images comparing pre- vs. post-TEMT (2 month) in single images of the cingulate cortex, with red, orange, and yellow pixels indicative of increased functional connectivity. Typically, only blue pixels (decreased functional connectivity) are present in AD subjects comparing fMRI scans separated by months. Reproduced with permission from Arendash et al., 2019 [52] and 2022 [51] publishers.

Demographics, Study Protocols, and Safety Monitoring. Our clinical work is based on a group of eight mild/moderate AD subjects (two males; six females) diagnosed with AD according to the National Institute of Neurological and Communicative Disorders and Stroke-Alzheimer’s Disease and Related Disorders Association (NINCDS-ADRDA) criteria. Subjects were at least 63 years of age (average 71 years), had an MMSE (Mini-Mental State Exam) score of 16–26 at screening, and an average ADAS-cog13 score of 36.7 at baseline. In addition, subjects had to have a Hachinski test score lower than 4, a Global Deterioration Score above 2, and confirmation of AD diagnosis through anatomic MRI scans, FDG-PET scans, and blood AD markers. Following attainment of baseline measures, all eight subjects were provided with 1-h TEMT sessions twice-daily and in-home (as administered by their caregivers) for 2 months. Following an ensuing 8-month period of no treatment, most of these subjects continued daily TEMT for an additional 4 months, followed by a second 5-month period of no treatment, and finally a 12-month period of daily TEMT—a total of 31 months (2½ years) from baseline to end of treatment for these individuals. Results being presented in this paper include a comprehensive analysis done for the first (2-month) treatment period [52,53] and analyses across all 31 months of treatment [51], including the 14–27 month period into treatment. Regarding safety monitoring over the 31 month treatment period, the primary safety measure was an Adverse Event Assessment implemented during every clinical visit. Associated safety measures involved the diaries kept by caregivers, which included frequent blood pressure/temperature measures from the subject, a checklist of whether activities were normal/abnormal, and the notation of any unusual behavior exhibited by the subject. A final safety measure involved anatomic MRI scans taken periodically during treatment. No adverse side-effects of TEMT were reported/observed for any patient on any of these safety measures through the 31-month treatment period. For the aforementioned eight AD subjects, we have published clear evidence that 2 months of daily TEMT can reverse their cognitive impairment (Figure 2A,B) and that extended TEMT can stop their cognitive/functional decline over a period of at least 2½ years (Figure 2C,D). More specifically, highly significant improvements in both ADAS-cog13 (4 points) and Rey AVLT recall (5 points) were present after 60 days of treatment (Figure 2A,B)—both of these cognitive reversals were maintained for at least 2-weeks after completion of treatment [52]. Over a treatment period of 31 months (2½ years), a “Cognitive Composite” score made up of eight measures from six tasks showed a complete stoppage of cognitive decline in AD subjects treated with TEMT during that period (Figure 2C). One example from these eight measures are MMSE scores that were maintained over the 2½ year treatment period by TEMT (Figure 2D). For comparison, another study that followed “untreated” AD patients with very similar demographics and baseline MMSE scores showed a continual, dramatic decline in MMSE scores over a 3-year period that averaged 4+ points per year (Figure 2D) [54]. Accompanying these benefits were changes in major blood and CSF markers for AD such as p-tau, t-tau, Aβ1-40, Aβ1-42, and oligomeric Aβ [51], along with functional brain fMRI changes indicative of TEMT-induced increases in functional connectivity between neurons in the brain (Figure 2E,F) [52].

Our pre-clinical and clinical studies have established at least three complementary mechanisms of TEMT action that appear to be “disease-modifying” against AD: (1) disaggregation of toxic Aβ and p-tau oligomers in the brain via vibrational disruption of hydrogen bonds connecting monomeric units [42,45,52], (2) enhancement of mitochondrial function in neurons via direct Complex IV activation [46], and (3) a rebalancing of both the peripheral (blood) and brain immune system effectors (e.g., cytokines) via several proposed mechanisms [53]. These mechanisms collectively appear responsible for stopping and reversing AD cognitive impairment in our clinical studies. Since most AD pathological processes occur “intraneuronally” [55], the ability of electromagnetic waves emitted by MemorEM devices to easily penetrate the cranium and neurons in all parts of the AD would seem critical for TEMT efficacy.

Our extensive pre-clinical studies predicted the aforementioned cognitive benefits in AD patients by showing that TEMT could both protect against and reverse cognitive impairment in AD transgenic mice—this, while limiting or reversing brain β-amyloid pathological development and enhancing brain mitochondrial function in the same AD mice [42,43,44,45,46,47]. Moreover, our pre-clinical cognitive and neuropathological findings in AD transgenic mice have been independently verified by at three international laboratories [56,57,58]. To our knowledge, no other groups have performed “clinical” TEMT studies.

In addition to AD, toxic protein aggregation mitochondrial dysfunction and/or immune dysregulation are primary features of most age-related diseases. Although TEMT could have wide-spread therapeutic applicability against these diseases through one, two, or all three of these mechanisms, we believe that rebalancing the immune system could be the most contributory mechanism for providing a decreased risk for age-related disease and increasing the human lifespan. This is because an imbalanced immune system primarily in favor of pro-inflammatory components appears to be central to numerous age-related diseases/conditions that manifest in the brain and/or body, such as Alzheimer’s Disease, Parkinson’s Disease, cardiovascular disease, osteoporosis, and rheumatoid arthritis [59,60,61,62,63]. If immune system imbalance is common to most of these age-related diseases, then a therapeutic that effectively prevents or treats one of these diseases (e.g., Alzheimer’s Disease) through immune/cytokine regulation could have therapeutic utility to many of them.

**Figure 3:**
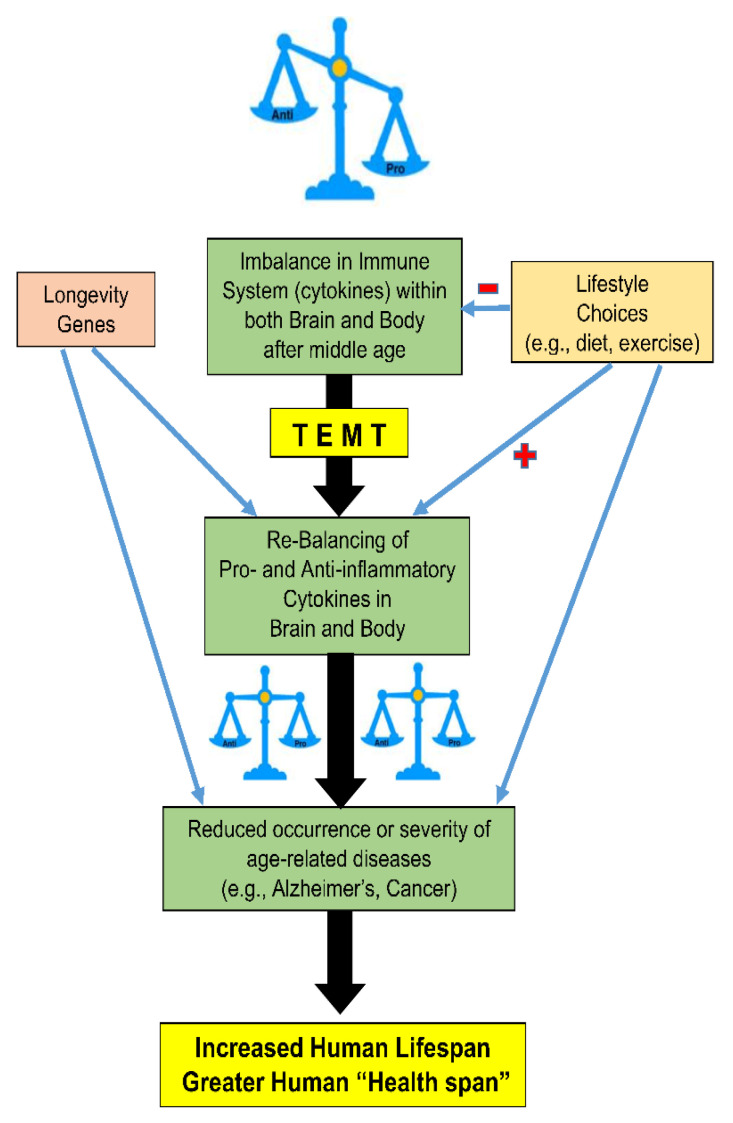
Rebalancing of the immune system by TEMT, proposed to result in fewer age-related diseases and ensuing increase in healthy lifespan.

In the remainder of this paper, we show that TEMT is capable of rebalancing a broad array of cytokines in the AD patient’s body (blood) and brain (CSF) to apparently re-establish the “balanced” immune system and lower inflammation of youth/middle age. We then provide likely mechanisms whereby TEMT exerts these cytokine-rebalancing effects to possibly extend the human health span. As shown in Figure 3, during the later decades of life, TEMT could complement good lifestyle choices or counter bad lifestyle choices by giving individuals a balanced immune system, perhaps irrespective of their lifestyle choices (e.g., exercise, smoking, drugs) and despite the cause of their immune imbalance.

## 4. TEMT Rebalances the Peripheral (Blood) Immune System in AD Subjects to Reduce Inflammation

Many AD researchers believe that AD, at least in part, is caused by a low-grade inflammation in the brain (29) and/or systemically in the blood [31,62,64,65]. However, anti-inflammatory agents, such as NSAIDs and prednisone, have failed to show clinical benefit in AD patients [66], as have agents purported to reduce brain microglial activity (ClinicalTrials.gov NCT02080364, NCT02916056; accessed on 19 April 2023). There is also evidence for AD as a disease involving “insufficient activation” of the immune system (low cytokine levels), resulting in an inability to resist AD pathogenesis and the progressive cognitive impairment it provides, especially in the Mild Cognitive Impairment (MCI) stage [67,68]. In either over-activated or under-activated scenarios, the immune system’s cytokine levels are imbalanced in the blood/brain and thus in need of “rebalancing” [20]. This has led to a new target for AD therapeutics being proposed—namely, a fine “rebalancing” of the immune system rather than trying to just suppress pro-inflammatory cytokines [20].

Given the sheer complexity of the brain and peripheral components of the immune system, wide-spread regulation of the immune system would appear to be a huge and most daunting task. However, a rebalancing of the brain and blood immune systems in AD subjects (as indexed by their effector cytokines) is exactly what we have shown to occur with daily TEMT [53]. TEMT resulted in a “rebalancing” in levels of 11 of 12 cytokines measured in blood plasma, which was associated with a reversal in cognitive impairment in the same AD subjects at the same time point (Figure 2A,B) [53].

**Figure 4:**
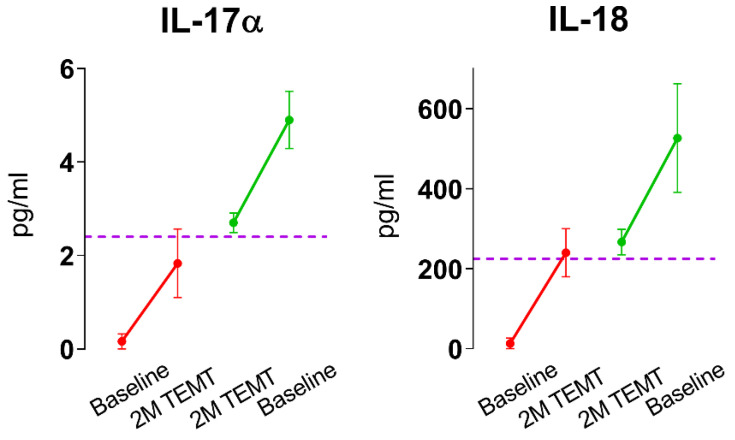
Two plasma cytokines in AD subjects, IL-17α and IL-18, showing a TEMT-induced “rebalancing” to normal aged levels, as indicated by the dotted purple line. If baseline plasma levels were low (below the purple dashed line), 2 months of TEMT resulted in an increase in plasma levels (red lines). Conversely, if baseline plasma levels were high, 2 months of TEMT resulted in decreased plasma levels (green line). In both cases, conversion toward normal age levels (purple dashed line) occurred. Means ± SEM. Reproduced with permission from Cao et al., 2022 [53] publishers.

Specifically, we found that after 2 months of twice-daily TEMT (1-h treatments), mild/moderate AD subjects exhibited a rebalancing of 11 of 12 cytokines in their plasma: GCSF, GMCSF, VEGF, PDGF, IL-8, IL-10, IL-15, IL-17α, IL-18, TGF-α, and IFN-γ [53]. *What is meant by a rebalancing in plasma cytokine levels?* After ranking all baseline levels for each cytokine and each AD subject, there was always a cut-off during ranking ascension, wherein the response to TEMT changed to the opposite direction. Those AD subjects with lower baseline levels for a particular cytokine (below the cut-off) showed increases in that cytokine after 2 months of daily TEMT. By contrast, those AD subjects with higher baseline cytokine levels in plasma for a particular cytokine (above the cut-off) showed treatment-induced decreases in plasma cytokines after 2 months of daily TEMT. AD subjects with baseline cytokine levels very near the cut-off showed little or no change following TEMT. Plasma cytokines IL-17α and IL-18 are presented as examples of this extraordinary rebalancing by TEMT in Figure 4. Thus, a gravitation to reported normal plasma cytokine levels occurred for 11 of 12 plasma cytokines after 2 months of daily TEMT.

C-Reactive Protein (CRP) is a protein made in the liver that is a widely accepted measure of systemic inflammation. CRP levels increase in the blood plasma when a condition (or conditions) is/are causing body inflammation. Indicative of the importance of inflammation during aging, higher CRP levels in aged individuals are strongly associated with a faster rate of cognitive decline over an 8-year period [69] and greater risk of impairment in Activities of Daily Living (ADL) [70]. CRP is increased in plasma from AD subjects [71,72] as well as being strongly associated with neurodegenerative inflammatory processes in AD brains [73]. Moreover, studies that followed non-disabled elderly patients for 4–5 years found that high plasma CRP levels predicted much greater risk of all-cause mortality [70,74], and a 23-year study found that high plasma CRP levels in older men predicted a shorter lifespan [75]. Thus, it appears that one factor on the pathway to healthy longevity is for aged individuals to maintain or achieve low plasma CRP levels (low peripheral inflammation) to reduce risk of cognitive decline, ADL impairment, and mortality itself.

**Figure 5:**
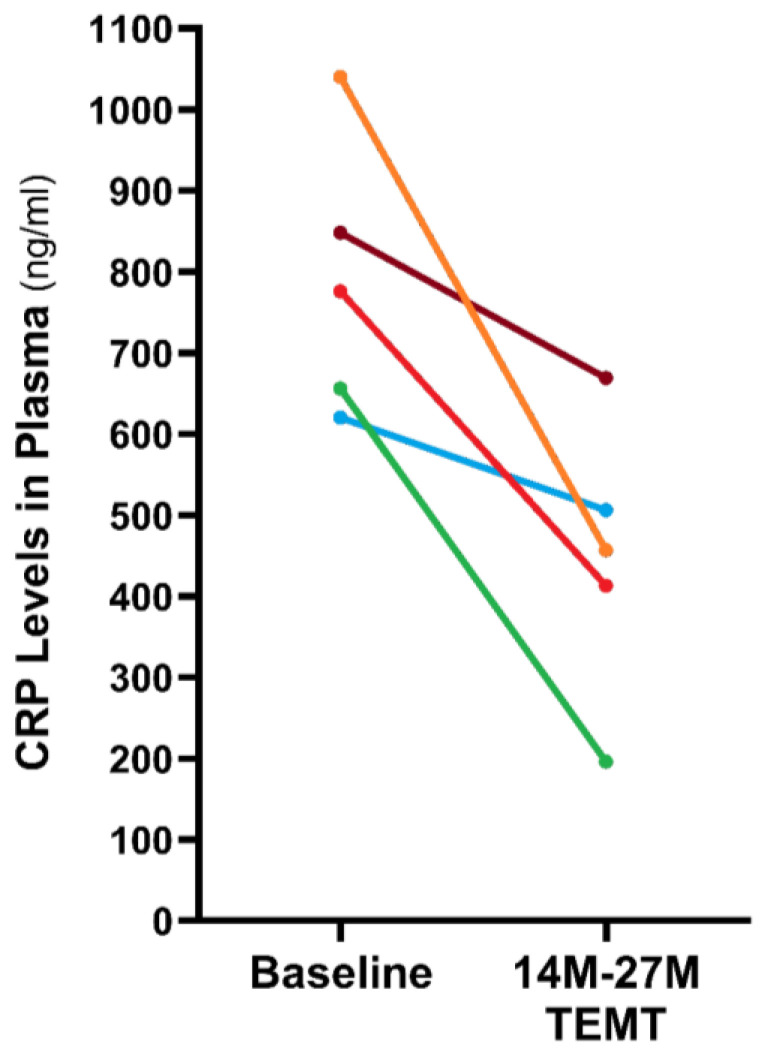
Much reduced plasma CRP in five AD subjects after a 14–27 month TEMT administration period. Reproduced with permission from Arendash et al., 2022 publishers [51].

We have measured CRP levels in plasma at both baseline and between 14 and 27 months into TEMT in AD subjects from our long-term treatment study (Figure 5) [53]. TEMT universally reduced CRP in plasma by an average of 43% (788 ± 75 ng/mL vs. 448 ± 76; *p* = 0.017). These decreases in plasma CRP levels, indicative of lowered peripheral inflammation, occurred in AD subjects who also showed stoppage of their cognitive decline over the same lengthy period [51]. Thus, the largely pro-inflammatory immune status in the plasma of AD subjects was substantially reduced by TEMT over a lengthy period.

We believe the same rebalancing will be the case for normal-aged individuals and those with other age-related diseases who present with a high level of inflammation (immune imbalance favoring pro-inflammatory cytokines) in their blood plasma.

## 5. Mechanism of TEMT-Induced Rebalancing of Human Peripheral (Blood) Cytokines

**Figure 6:**
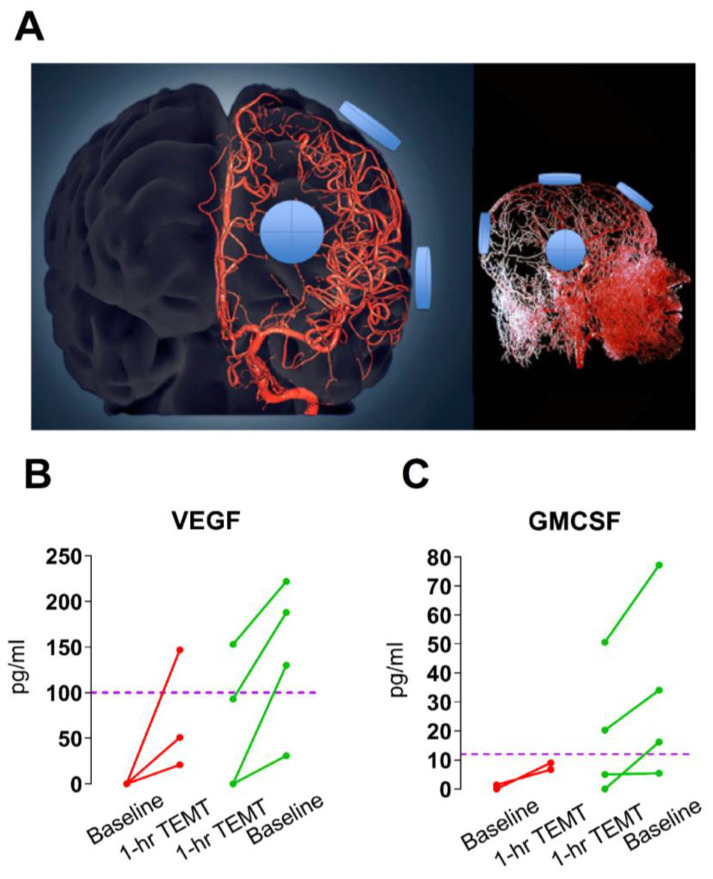
(**A**) Three-dimensional model of the cerebral arterial tree showing the location of four EMF emitters on the surface of the head’s right side and in close proximity to affect all components within the cerebrovascular tree. (**B**,**C**) Effects of a single 1-h TEMT treatment on plasma levels of the cytokines VEGF and GMCSF in AD subjects. Red and green lines indicate subjects that had low and high levels, respectively, of these cytokines at baseline. A rebalancing of cytokines is already evident after this single TEMT treatment. Normal-aged levels are indicated by the dotted purple line. Reproduced with permission from Cao et al., 2022 [53] publishers.

First, the ability of TEMT to impact cytokine levels in the systemic vasculature is very likely due to direct effects of electromagnetic (radiofrequency) waves on blood flowing through the head and brain, underneath the eight EMF emitters (Figure 6A). Secondly, the ability of TEMT to rebalance cytokines in the blood does not take days or months to manifest itself. Strikingly, it is already becoming evident after a single initial 1-h TEMT to AD subjects [53]. This rapid rebalancing in blood cytokines is shown in Figure 6B,C for the cytokines VEGF and GMCSF. Although there may be other possible explanations for this quick TEMT-induced rebalancing action on blood cytokines, we believe the most likely mechanism involves red blood cells (RBCs).

**Figure 7:**
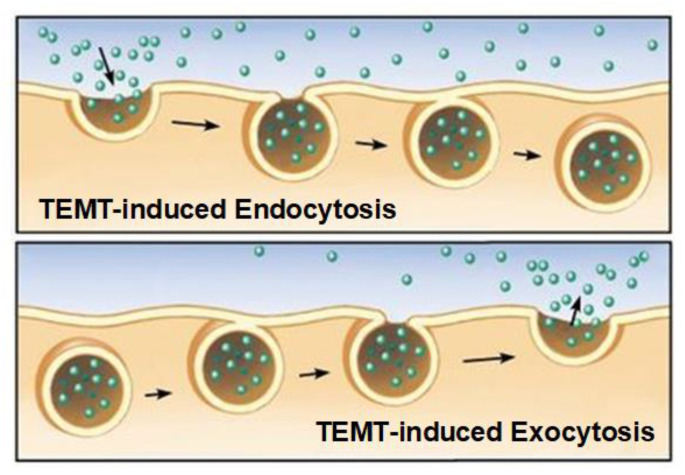
The two mechanisms of RBC membrane trafficking, exocytosis and endocytosis. TEMT is proposed to increase this membrane trafficking via increased membrane fluidity, resulting in flux of a given cytokine in or out of RBCs depending on their plasma concentration.

RBCs highly concentrate cytokines, resulting in RBC concentration gradients with plasma that normally average 12:1 for 46 cytokines from healthy volunteers [76]. This is important, because TEMT exposure to human RBCs going through the head/brain vasculature appears to cause dielectric loss in RBC membranes to lessen their membrane dipole–dipole interactions [77] through γ-dispersion [78]. As such, the resultant rotational motion of membrane water dipoles would increase membrane trafficking (endocytosis and exocytosis), such as for blood cytokines. A good example of this proposed TEMT-induced increase in membrane trafficking/permeability is a study wherein RBCs were exposed to electromagnetic waves at 18 GHz for several one-minute treatments [79]. This treatment induced RBC uptake (endocytosis) of nanospheres that were initially present only in the medium. Shortly following the 1-min treatments, nanospheres were observed both passing through the RBC cell membrane and already internalized. Such RBC membrane trafficking by both endocytosis and exocytosis is diagramed in Figure 7.

We have obtained evidence for this proposed EMF-induced increase in RBC membrane transport/fluidity from RBCs in blood from our AD subjects who were given 2 months of daily TEMT [53]. This evidence involves the fact that over 99% of α-synuclean (α-syn) in blood is in RBCs, with less than 1% being in other components of blood [80]. As such, human plasma samples typically do not contain measurable amounts of α-syn. This was the case for four of our AD subjects evaluated at baseline for α-syn. However, after a single 1-h TEMT, two of these four AD subjects showed measurable α-syn levels in plasma (3500 and 6250 pg/mL) and by Day 7 into treatment, three of the four AD subjects showed α-syn levels in plasma averaging 4800 pg/mL immediately following treatment. By the end of daily treatment on Day 60, plasma α-syn levels in three of the four subjects averaged 92,000 pg/mL. However, after an ensuing post-treatment period of 2 weeks, levels returned to undetectable levels in all of these subjects. This post-treatment up-take of α-syn was presumably due to re-establishment of the normal RBC-to-plasma ratio of over 99:1.

Thus, we propose that TEMT administered to the human head via our MemorEM devices increases the fluidity or membrane trafficking of RBCs in blood vessels going through the head/brain, resulting in cytokines going up or down their RBC–plasma concentration differences. Therefore, if a given cytokine’s concentration in plasma is lower than normal, a net flux of that cytokine across RBC membranes and out of RBCs occurs, with just the opposite net flux occurring if plasma concentrations are too high. This would account for TEMT’s ability to rapidly modulate/normalize plasma cytokine (or α-syn) concentrations in systemic blood.

We further postulate that one important reason centenarians live so long with a balanced immune system is because they preserve the membrane fluidity of their RBCs, thus maintaining RBC-to-plasma cytokine levels to those of young adulthood/middle age. Indeed, nonagenarians and centenarians have increased RBC membrane integrity and fluidity compared to the general population [81,82,83]. In that capacity, TEMT could be “rejuvenating” RBC membrane integrity and fluidity in aged individuals, with an ensuing balance of the systemic immune system that provides protection against age-related diseases and increased healthy longevity. In essence, TEMT could be acting like a continual blood transfusion from younger individuals to rejuvenate the blood of older/diseased individuals, resulting in reduced systemic inflammation and an increase in lifespan. In this regard, the process of “heterochronic parabiosis” to join the systemic blood systems of old mice to young mice has rejuvenating effects on various tissues in the old mice by reducing their blood levels of pro-inflammatory cytokines [84,85], Relatedly, heterochronic parabiosis studies have shown an increase in lifespan for the old mice [86].

## 6. TEMT Rebalances the Human Brain’s Immune System in AD Subjects to Reduce Inflammation

**Figure 8:**
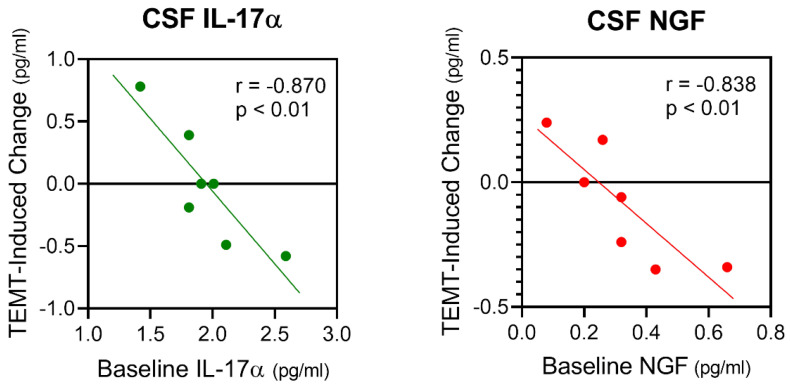
Two CSF cytokines in AD subjects, IL-17α and NGF, showing a TEMT-induced “rebalancing”, with direction and extent of change linked to cytokine baseline levels. Note highly significant correlations. Reproduced with permission from Cao et al., 2022 [53] publishers.

Cerebrospinal fluid (CSF) can provide an excellent index of cytokine concentrations and the extent of inflammation in the brain’s neurons and parenchyma. As such, we have taken CSF samples from the AD subjects in our studies to gain insight into potential TEMT-induced changes in both brain cytokine concentrations and general brain inflammation [53]. For five of seven cytokines measurable in CSF (IL-17α, NGF, GCSF, VEGF, TGFα), the response to 2 months of daily TEMT was entirely dependent on baseline CSF levels—just as was the case for plasma cytokines from these same AD subjects (Figure 4). Specifically, TEMT increased a given CSF cytokine if lower levels were present at baseline, while TEMT decreased a given CSF cytokines if higher levels were present at baseline. Two examples of this rebalancing are shown in Figure 8 for IL-17α and NGF. Even for the remaining two measurable cytokines in CSF (IL-8 and IL-15), the TEMT response from all but one AD patient was dependent on whether baseline cytokine levels were lower or higher. Thus, a 2-month period of daily TEMT can rebalance 11 or 12 cytokines in plasma and essentially all 7 cytokines in brain/CSF.

**Figure 9:**
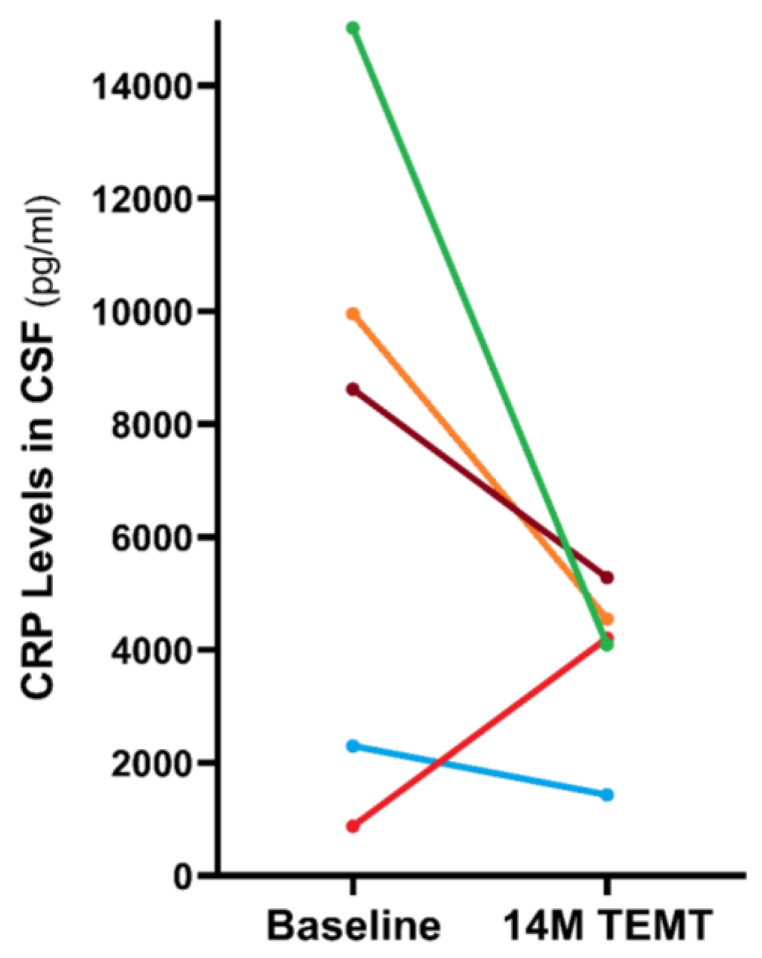
Reduction in overall brain inflammation after 14–27 month TEMT period, as indexed by CRP, in four of five AD subjects. The one subject showing increased CRP had a rebalancing of very low baseline levels. Reproduced with permission from Arendash et al., 2022 [51] publishers.

Just as was done for determining the level of general inflammation in the periphery with CRP measurement (Figure 5), we compared CSF samples taken at baseline to those taken at 14 months into TEMT for CRF levels, as a general index of brain inflammation. TEMT reduced CRP levels in four of the five subjects compared to their baseline by 51% (Figure 9). The one AD subject that displayed a TEMT-induced increase in CRP levels had very low baseline levels, suggesting that a rebalancing of CRP in the CSF/brain had occurred. Thus, TEMT appears to have reduced the overall pro-inflammatory immune status of AD subjects in both the brain/CSF and the periphery, and over an extended period. Indeed, this reduced inflammation alone could be responsible for much of the long-term cognitive stability provided to AD subjects by TEMT [51].

## 7. Possible Mechanisms of TEMT-Induced Rebalancing of Human Brain/CSF Cytokines

The mechanism(s) responsible for TEMT-induced rebalancing of brain/CSF cytokines and reduction in brain overall inflammation is less clear than that for the TEMT-induced rebalancing in systemic blood. Although it is possible that plasma cytokine levels could influence CSF cytokine levels or vice versa, this would appear to be unlikely because there were no correlations between baseline plasma and baseline CSF cytokine levels. Moreover, baseline CSF levels of four cytokines (GCSF, IL-8, IL-15, and TGFα) were much higher than their baseline plasma counterparts for all AD subjects. Thus, cells within the brain were secreting at least these four cytokines and TEMT affected the brain secretion of all of them.

Exactly how TEMT affects brain cytokine secretion, and from which brain cell type(s), is currently unknown. Microglia, as well as astrocytes, are known to secrete cytokines [87] and prior studies have reported EMF (electromagnetic field) effects on both cell types. Specifically, rats exposed to EMF at 915 MHz exhibited microglial cell activation thereafter [88], and activation of astrocytes in cell culture has been reported at 900 MHz EMF [89,90]. However, no effect of 900 MHz EMFs at similar power levels was reported on the release of several cytokines from astrocyte cell cultures or on microglial activation in culture [91]. An alternative, and perhaps more likely, mechanism of CNS/CSF cytokine regulation by TEMT may be by affecting choroid plexus epithelial cells or resident macrophages within the choroid plexus [92,93]. Choroid plexus epithelial cells have been shown to produce and secrete cytokines into the CSF [92]. Aquaporin-1 channels in their apical walls can not only regulate water transport, but can also act as gated ion channels [94]. In this regard, Aquaporin channels have been shown to be regulated by an electrostatic potential [95], which is consistent with their modulation by electric fields generated by TEMT in the brain. Whether microglial, astrocytes, and/or choroid plexus epithelial cells are modulated by TEMT in providing a “rebalance” of multiple cytokines in the CSF and/or brain is an open question.

## 8. Potential Human Benefits of Rejuvenating the Balance in Cytokine Levels within Blood and Brain

An obvious and potentially huge benefit of a TEMT-induced rebalancing of cytokine levels within blood and/or brain is a much-reduced risk or severity of age-related diseases and an ensuing increase in healthy longevity. Given the near-universal involvement of immune dysfunction in diseases of aging [25,26], our expectation is that this **single** therapeutic intervention of TEMT may be an all-encompassing therapeutic bridge to healthy aging and longer lifespan. In view of the results described herein from our completed clinical studies, TEMT is clearly a frontrunner in gerotherapeutic interventions, especially with its apparent ability to stop/reverse progression of a primary disease of aging (i.e., AD), and possibly doing so through an immune-rebalancing mechanism applicable to many age-related diseases and healthy longevity itself. As an example of the latter, many centenarian males have a genotype associated with high production of IL-10, an anti-inflammatory cytokine that strongly counters pro-inflammatory processes [96,97]. In our 2-month TEMT treatment study [53], plasma IL-10 levels in male AD subjects were low at baseline, but increased by over 100% by the end of treatment. This suggests that long-term continuation of TEMT would maintain or elevate even further this cytokine so very important for male longevity.

**Figure 10:**
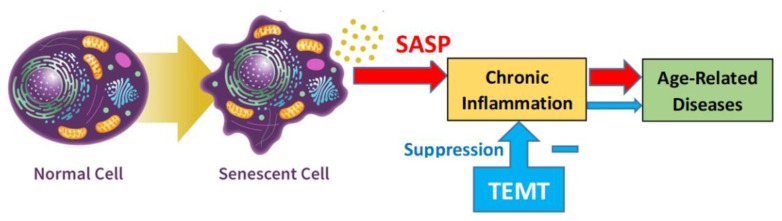
By rebalancing cytokines in both brain and body in aged individuals, TEMT would greatly decrease chronic inflammation (including SASP-induced inflammation) to reduce age-related diseases and increase heath span. Thus, removal of SASP by senolytic drugs may not be needed to decreased risk or occurrence of age-related diseases.

A second potential benefit, linked directly to TEMT’s ability to rebalance cytokines and to dramatically reduce overall inflammation in the brain/CSF and peripheral blood, involves the age-related transformation of normal cells into senescent cells within body and brain—cellular senescence (Figure 10). These senescent cells have undergone widespread changes in protein expression/secretion during aging, resulting in their production of an inflammatory cocktail called the Senescence-Associated Secretory Phenotype or SASP (Figure 10). Among SASP’s components are interleukins and cytokines such as IL-6, GCSF, GMCSF, and VEGF [11]. As previously discussed, senolytic drugs are being developed to kill such senescence cells in hopes of reducing their SASP inflammatory products in body and brain [16]. However, if TEMT can rebalance/counter the pro-inflammatory interleukins/cytokines secreted by SASP as part of its overall cytokine rebalancing to decrease chronic inflammation, then senolytic drugs may not be needed, or at best would be used as an adjunct gerotherapeutic.

Parenthetically, trying to rid the “brain” of senescent neurons with senolytics could cause a continual, massive activation of brain microglia/phagocytes attempting to get rid of debris from dead senescent neurons that could actually worsen brain inflammation long-term. Along this line, a just-completed study involving the administration of the senolytics Dasatinib+Quercetin (D+Q) to AD patients for 3 months reported an increase in plasma levels of the pro-inflammatory cytokine IL-6 and a decrease in brain/CSF levels of the anti-inflammatory cytokine IL-10, both suggesting that an increase in brain and/or peripheral inflammation may have been induced by DQ treatment [14].

## 9. General Discussion

There is substantial evidence that most diseases in older age involve inflammation in the brain and/or body due to a chronic over-activation of pro-inflammatory cytokines over anti-inflammatory cytokines (“inflamm-aging”) [25,26]. By contrast, individuals in young adulthood and middle age have an immune system balanced in these two components, as do many centenarians (100+ years), effectively providing immune protection from environmental and body insults. We have therefore proposed that a gerotherapeutic that can “rebalance” pro- and anti-inflammatory cytokines in aged individuals will reduce the risk or severity of age-related diseases and increase healthy longevity. This perspectives paper provides clinical evidence that such a “rejuvenating” gerotherapeutic is not a drug, but rather a bioengineered medical device that provides Transcranial Electromagnetic Wave Treatment (TEMT), the *MemorEM*. In that regard, TEMT administration to aged/diseased individuals could be comparable to them receiving continual blood infusions from young adult or middle-aged individuals who have a balanced (youthful) immune system in their blood—the result being much reduced systemic inflammation and increased longevity through TEMT [84,85,86].

The results presented, and conclusions reached in this paper are based on our three published clinical papers involving the same mild/moderate AD subjects given TEMT for a period of 2½ years and evaluated at 2 months, and 14–31 months into that period [51,52,53]. At 2 months into treatment, analysis of 11 of 12 cytokines in blood and all 7 cytokines in brain/CSF revealed an extraordinarily consistent ability of TEMT to “rebalance” the largely pro-inflammatory status of the immune system in these subjects [53]. Specifically, if a given cytokine’s level was high in blood plasma or CSF, TEMT reduced its levels to normal aged individuals, and vice versa if they were low. The mechanism for blood rebalancing of cytokines by TEMT appears to involve the unique ability of RBCs to concentrate cytokines [76] and to respond to electromagnetic waves by increasing their membrane fluidity [79] to rebalance plasma cytokines with an appropriate flux of cytokines in or out across their membranes. We believe that the same rebalancing of blood/plasma cytokines by TEMT will occur in both normal aged and age-diseased individuals. This is because those cytokine levels in individual subjects that were near normal levels at baseline did not show a TEMT-induced change in their levels thereafter.

Inasmuch as many studies have linked immune/cytokine imbalance to the “progressive cognitive decline” seen in AD (a foremost disease of aging), AD serves as a good initial test for the ability of TEMT to impact this cardinal characteristic of AD. Indeed, AD subjects given 2 months of daily TEMT showed a reversal of their cognitive impairment in several primary measures (e.g., ADAS-cog13, Rey AVLT) [52]. During a 2½ year period of treatment, these AD subjects showed stable cognitive performance compared to baseline—a stoppage of cognitive decline [51]. Moreover, levels of key AD markers (e.g., p-tau, t-tau, Aβ1-40, Aβ1-42, oligomeric Aβ) in both plasma and CSF/brain were affected by TEMT either through outright reductions or rebalancing [51]. Although the other two mechanisms of TEMT action (brain mitochondrial enhancement and brain disaggregation of toxic protein oligomers) probably contributed to the cognitive benefits and AD marker changes induced by TEMT, the rebalancing of both body and brain cytokines by TEMT may have played a more prominent role. In any event, the rebalancing of cytokines and consequent decrease in overall brain and blood inflammation provided by TEMT may negate the need for senolytic drugs such as Dasatinib+Quercetin or Fisetin. This is because TEMT could probably neutralize the pro-inflammatory products in SASP secretions from senescent cells as part of its overall rebalancing of brain and body/blood cytokines.

The field of gerotherapeutic drug development has continued to advance pre-clinically, although only a few human clinical trials have been initiated or completed. In that regard, a small controlled clinical trial administering Rapamycin to healthy aged subjects for 8 weeks did not have any effect on cognitive performance, immune endpoints, or self-perceived health [10]. Regarding the senolytics Dasatinib+Quercetin (DQ), an open label clinical trial in pulmonary fibrosis patients found no effect on measures of pulmonary function and wide-spread side effects of skin irritation/bruising and nausea/heart burn [98]. Another clinical trial of DQ in patients with diabetic kidney disease just evaluated senescent cell markers, with no endpoints for diabetic kidney disease [99]. There are also on-going clinical trials of DQ in osteoporosis (ClinicalTrials.gov NCT04313634; accessed on 22 April 2023) and the senolytic Fisetin in osteoarthritis (ClinicalTrials.gov NCT04210986; accessed on 22 April 2023). Most recently, a 3-month clinical trial of DQ in five early AD patients failed to show any cognitive benefits of treatment and, in fact, resulted in significant or near-significant declines in several cognitive tasks. The study also reported evidence of increased brain/peripheral inflammation (higher IL-6 and lower IL-10 levels), a significant elevation in total blood cholesterol, and no treatment effect on plasma or brain AD markers [14].

Methformin is the most clinically-investigated gerotherapeutic due to its prominence in the treatment of Type II diabetes. The purported effect of Methformin to increase the health span in humans appears to result from its anti-hyperglycemic and insulin-sensitizing effects, which then secondarily contribute to a possible reduced risk of age-related diseases and increased health span [6]. For example, Methformin’s reduction in some pro-inflammatory cytokines is indirectly through reduction in NF-kB [100] and therefore not a specific anti-inflammatory action of the drug. Results from prospective and randomized controlled clinical trials evaluating cancer risk with Methformin have been mixed at best [6], and data related to its effect on risk of neurodegenerative diseases such as Alzheimer’s Disease remain ambiguous [101]. Thus, continued controversy is present as to whether or not Methformin reduces risk of age-related diseases and increases health span. Table 1 compares Methformin to TEMT as perhaps the two leading clinical-stage gerotherapeutics purported to increase healthy longevity.

There are a number of caveats that should be mentioned relative to our clinical data postulating that TEMT may be a rejuvenating gerotherapeutic that can reduce the risk of age-related diseases and increase healthy longevity. A first caveat involves the attainment of all our human immunological results from the blood and brain/CSF of AD subjects. It is therefore possible that a rebalancing of the immune system will not be seen in normal (non-AD) subjects or in those with other age-related diseases whose immune systems are out-of-balance. However, the fact that AD subjects whose baseline plasma cytokine levels were near normal-aged levels did not show a sizable TEMT-induced increase or decrease away from this average level advocates that TEMT’s rebalancing of the immune system’s cytokines will occur irrespective of the subject being non-diseased or having an age-related disease/condition.

A second caveat engages the point that, if TEMT is a true gerotherapeutic, it will need to be verified as beneficial for a variety of diseases whose major commonality is that they are all age-related diseases. To that end, we are planning to initiate a longitudinal 6-year double-blind, controlled clinical trial in aged individuals (the TEMT during Aging [TEMTA] trial) to determine if long-term TEMT can: (1) reduce the occurrence and severity of age-related diseases during that lengthy period, and (2) reverse the inflammatory age of participants, as determined by CRP levels and/or new “inflammatory clocks of aging” that have recently become available [23]. If fewer age-related diseases and/or reversal of inflammatory aging is observed in this first clinical study to comprehensively evaluate a gerotherapeutic intervention long-term, evidence will have been attained that TEMT may indeed increase healthy longevity. Indeed, determining if TEMT reduces inflammatory age will probably take only a year or two of TEMT.

A third caveat is that TEMT may be affecting other components of the peripheral immune system besides RBCs. Along this line, no effects of 900 MHz EMF (RF) have been found on the immune system (both T- and B-cell compartments) [102]. Specifically, with 900 MHz RF treatment for one month to mice, neither T- nor B-cell compartments were affected [103]. Similarly, the same one-month treatment protocol did not affect the B-cell peripheral compartment (T1 and T2 cells, mature follicular and marginal zone B-cells) or antibody (IgM and IgG) production in mice [104]. In addition, no effects of 900 MHz RF on mouse bone marrow cells were reported on their long-term (3-month) ability to reconstitute peripheral T and B cells, and no differences were found in thymocyte number, frequency, or proliferation [105]. Although, at a higher 1950 MHz, in vitro studies involving human lymphocytes and monocytes have reported no EMF effects [106]. Thus, there does not appear to be any evidence to date that EMFs around 900 MHz effect cellular components of the peripheral immune system. However, because no studies have utilized Primary Cells of the Immune System (PBMCs), we are currently evaluating EMF-treated PBMC cell cultures (T-cells, B-cells, stem cells, NK-cells, and DC-cells) for pro- and anti-inflammatory cytokine levels, and other immune markers.

A final caveat involves the limited number of subjects (5–8) in our clinical work, and there not being a control/placebo group. To this point, the need for highly (clinically) significant effects to be present in such a small group and our consistency in demonstrating those effects argue that they are truly significant, and that a larger, controlled study will confirm the effects we have reported. Accordingly, we are initiating a controlled double-blind Phase IIb/III clinical trial in which mild/moderate AD subjects will be given TEMT or placebo for 12 months. The FDA in the U.S. has provided important design suggestions for this trial under the “breakthrough” designation they have bestowed upon NeuroEM Therapeutics and its TEMT technology.

Before the decline in lifespan induced by COVID-19 beginning in 2020, there were already a number of developed countries wherein lifespan had not increased by much, if any, over the last 1–2 decades (e.g., United States, England, Italy, Russia) [107,108]. An increased incidence of age-related diseases would appear to be the main factor for this recent absence of a continued increase in human longevity in some countries, particularly in concert with bad lifestyle choices. We propose that TEMT may be able to, at least in part, circumvent such harmful lifestyle choices through its ability to rebalance both brain and body cytokines. Although it would be more convenient to take a pill for increasing healthy longevity, the *MemorEM* device we have developed may only be needed periodically (e.g., weekly, bimonthly) after an initial period of daily in-home treatments for some months. This TEMT paradigm would seem to be of minimal inconvenience for attaining a balanced immune system that could reduce the risk/severity of age-related diseases and increase healthy longevity.

For maximal convenience, it is conceivable that TEMT’s emitters could be implanted subcutaneously on the cranium or positioned directly on the brain’s surface via cranial windows. In either scenario, automatic and emitter activation by local circuitry/chips located in or outside the body is envisioned to provide brain TEMT in an individualized, changeable fashion—in essence providing a brain “neuro-interface” ability for TEMT administration, in a similar way that Elon Musk’s company *Neurolink* is developing brain-implantable chips via cranial windows to provide electrical stimulation to neurons via many thin electrodes.

Since most age-related diseases are characterized by immune imbalance (usually in favor of pro-inflammatory cytokines), TEMT’s ability to rebalance many cytokines of the brain and body’s immune system could reduce the occurrence and/or severity of these diseases—and, in so doing, may quite feasibly provide humans with a “Fountain of Healthy Longevity”. Put another way, if most age-related diseases have the commonality of immune dysregulation, and if a major disease of aging (i.e., Alzheimer’s) has its immune dysregulation corrected by TEMT in concert with the removal of that disease’s major symptom (i.e., progressive cognitive decline), then it is reasonable to expect that TEMT could successfully treat the immune dysregulation of other age-related diseases. Resolution of this premise awaits further clinical trials with TEMT in aged individuals, as exemplified by the aforementioned TEMTA study.

## 10. Conclusions

In conclusion, our clinical studies suggest that TEMT is a gerotherapeutic medical device with real potential across the broad realm of age-related diseases bearing immune imbalance. It is proposed that reducing the risk of, or treating, these diseases through a TEMT-induced rebalancing of the immune system could provide the longer and heathier lifespan experienced by centenarians and that most of us strongly desire.

## 11. Patents

Three patent applications have resulted from the research presented in this review, as submitted by NeuroEM Therapeutics, Inc.: in the U.S. as CIP US-2022-0040492-A1 and CIP US 17/975,820, while internationally as PCT/US21/56328.

## Figures and Tables

**Table 1:** A Comparison of TEMT with Methformin as Gerotherapeutics.

Methformin	TEMT
Reduces only pro-inflammatory cytokines in	Rebalances both pro- and anti-inflammatory
blood (no effect on anti-inflammatoy cytokines)	cytokines in both blood and brain
Decreases CRP only in blood	Decreases CRP in both blood and brain
Has side effects and some reports show an	No long-term side effects found so far, but is new
increased risk of AD and PD	and has not been investigated much clinically
Approved only for type 2 diabetes, but may	Not approved for any indication so far, but has
have other disease indications	FDA “breakthrough” designation for AD
Old drug; much investigated	New bioengineered technology
Two mechanisms of action against aging	Three mechanisms of action against aging
Anti-hyperglycemic/greater insulin sensitivity Can reduce some pro-inflammatory cytokines	Rebalances immune system’s cytokines in both blood/body and brain Enhances mitochondrial energy production in the brain Disaggregates toxic protein oligomers in brain that are involved in many diseases of aging

## Data Availability

The datasets used and/or analyzed during the current study are available from the corresponding author upon reasonable request.
